# Gut Microbiome-Liver-Brain axis in Alcohol Use Disorder. The role of gut dysbiosis and stress in alcohol-related cognitive impairment progression: possible therapeutic approaches

**DOI:** 10.1016/j.ynstr.2025.100713

**Published:** 2025-02-08

**Authors:** Emilio Merlo Pich, Ioannis Tarnanas, Patrizia Brigidi, Ginetta Collo

**Affiliations:** a Gelf Health, Milano, Italy; b Trinity College Dublin, Global Brain Health Institute, Dublin, Ireland; c Altoida Inc., Washington DC, USA; d Human Microbiomics Unit, Department of Medical and Surgical Sciences, University of Bologna, Italy; e Human Neuropharmacology Unit, Department of Molecular & Translational Medicine, University of Brescia, Italy

## Abstract

The Gut Microbiome-Liver-Brain Axis is a relatively novel construct with promising potential to enhance our understanding of Alcohol Use Disorder (AUD), and its therapeutic approaches. Significant alterations in the gut microbiome occur in AUD even before any other systemic signs or symptoms manifest. Prolonged and inappropriate alcohol consumption, by affecting the gut microbiota and gut mucosa permeability, is thought to contribute to the development of behavioral and cognitive impairments, leading to Alcohol-Related Liver Disorders and potentially progressing into alcoholic cirrhosis, which is often associated with severe cognitive impairment related to neurodegeneration, such as hepatic encephalopathy and alcoholic dementia.

The critical role of the gut microbiota is further supported by the efficacy of FDA-approved treatments for hepatic encephalopathy in alcoholic cirrhosis (i.e., lactulose and rifaximin). To stimulate new research, we hypothesize that interactions between a maladaptive stress response and a constitutional predisposition to neurodegeneration underlie the progression of AUD to conditions of Alcohol-Related Clinical Concerns with severe cognitive impairment, which represent a significant and costly burden to society. Early identification of AUD individuals at risk for developing these conditions could help to prioritize integrated therapeutic interventions targeting different substrates of the Gut Microbiome-Liver-Brain axis. Specifically, addiction medications, microbiome modulators, stress-reducing interventions, and, possibly soon, novel agents that reduce hepatic steatosis/fibrosis will be discussed in the context of digitally supported integrated therapeutic approaches. The explicit goal of this AUD treatment performed on the early stage of the disorder would be to reduce the transition from AUD to those conditions of Alcohol-Related Common Clinical Concerns associated with severe cognitive impairment, a strategy recommended for most neurological neurodegenerative disorders.

## 1 The gut microbiome-liver-brain axis and neurodegeneration in alcohol-related disorders

The 'Gut-Liver-Brain axis' is a recently established construct that interconnects these organs in a complex homeostatic system, contributing to growth, maintenance, and survival throughout the lifespan, as well as in response to pathological dysfunctions and stressors (Carabotti et al., 2015; Patel et al., 2016, Rusch et al., 2020). With advancements in metagenomic techniques, the gut microbiota has gained prominence within this construct, leading some researchers to propose the updated term of 'Gut Microbiome-Liver-Brain' axis (Cryan et al., 2019; Margolis et al., 2021; Smith et al., 2024).

Alterations in the gut microbiota have been implicated in Alcohol Use Disorder (AUD), a reversible condition characterized by recurrent, excessive, and uncontrolled alcohol consumption episodes, typically followed by withdrawal periods marked by craving, anxiety, and depression (NIAAA Alcohol Use Disorder, 2024, Horrell et al., 2022). According to the DSM-5, AUD is defined as a syndrome of sustained problematic alcohol use and clinically significant impairment, diagnosed as mild, moderate, and severe, based on the number of symptoms out of the 11 criteria assessing behavioral and physical manifestations and recorded over the last 12-month period (DSM-5, DSM-5 TRTM, 2024). Chronic problematic use of alcohol over the years can lead to clinical complications, also defined as "Common Alcohol-Related Clinical Concerns” which include pathological damage of the liver, intestine, pancreas, lungs, cardiovascular system, immune system, endocrine system, peripheral nerves, and in the brain (Rehm et al., 2010; NIAAA Core Resource on Alcohol, 2024). The definition here used of "Alcohol-Related Clinical Concerns” was proposed based on the current NIAAA-NIH terminology, which better characterizes the conditions previously included in the more general term ‘alcoholism’ and, indirectly, refers to the updated CDC classification which defines the Alcohol-Related Disease Impact based on 17 ICD10-CM different codes in 9 different organs/systems (CDC, Alcohol Related Disease Impact, 2024).

In this review, we will conceptualize problematic alcohol use as a continuum from *mild AUD*, generally in younger individuals, to severe *AUD with Alcohol-Related Clinical Concerns* in older individuals (e.g., cirrhosis, pancreatitis, hepatic encephalopathy, etc., including cancer), with uncontrolled alcohol drinking habits being the common behavioral pathogenetic factor during the development of the disorder.

Given the clear occurrence of pathological alterations in the Gut Microbiota-Liver-Brain axis in individuals with *AUD with Alcohol-Related Clinical Concerns* over the years, we hypothesized that these pathological effects begin at the onset of AUD conditions, initially affecting the gut microbiota and brain, even before the development of Alcohol-Related Lived Disease (ARLD)-steatosis. The rapid, massive and repeated exposure of the gastrointestinal tract to high concentrations of alcohol would first affect the composition of the gut microbiota and the functions of the intestinal mucosa, and, after being adsorbed into the body, the resulting high concentrations of alcohol would differentially affect the visceral organs, e.g., intoxicating in the brain, but, initially, minimally affecting the liver. The evidence supporting early damage to the gut and gut microbiome in individuals with AUD are summarized in Chapter 3.

A second implication of the conceptualization of a pathogenetic continuum from *mild AUD* in young adult individuals to *AUD with Alcohol-Related Clinical Concerns* in elderly adults is that the neurodegenerative effects of alcohol in the brain would build progressively, with a marked effects in a subgroup of individuals. In other words, we surmise the possible existence of a constitutive liability to neurodegeneration in selected individuals that would show cognitive impairment and neuroimaging evidence of neurodegeneration already at the initial stage of *mild to moderate AUD,* eventually evolving to *AUD with ARLD and severe alcohol-related neurologial syndromes,* e.g., *Hepatic Encephalopathy* or *alcoholic dementia*. The concept of pre-morbid risk in selected individuals is already well accepted for prototypical neurodegenerative disorders such as Alzheimer's or Parkinons's disorders. The supporting findings regarding individuals with AUD are described in Chapter 4.

A third element of critical relevance that we proposed here, is the concurrent exposure to stress that would affect the Gut Microbiome-Liver-Brain axis, resulting in an amplification of the alcohol-related damage, since stressors and alcohol share several pathogenic factors. In Chapter 2 we briefly listed the main common pathogenetic factors, while in Chapter 5 we summarized the findings supporting the role of stress in AUD. Finally, in Chapter 6, we explored potential integrated therapeutic interventions targeting the Gut Microbiome-Liver-Brain axis within the AUD continuum.

**Fig. 1:**
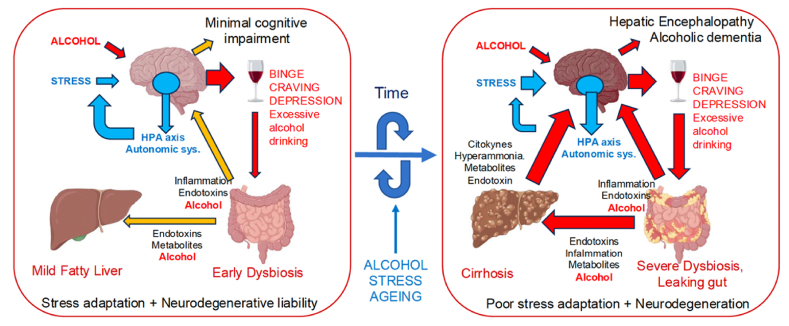
The cartoon illustrates the proposed "working hypothesis” regarding the role of the Gut Microbiome-Liver-Brain axis during the progression from the early stages of Alcohol Use Disorder (AUD) (left panel) to the more advanced stages of *AUD with Alcohol-Related Clinical Concerns* (right panel) when exposed to stressors. **Left panel.** Recurrent ethanol exposure associated with binge drinking initially affects gut microbiota composition, gut barrier permeability, and neurological control of reward/negative emotion, craving, and decision making, contributing to addiction. These effects are achieved by triggering pathogenic processes that alter the underlying cellular substrates in various target organs of the gut microbiota-liver-brain axis, including direct toxic effects of alcohol, increased gut permeability, dysbiosis, bacterial endotoxins, and proinflammatory mediators. At this stage the brain's stress response circuitry is still functional, triggering adrenal and autonomic system response, providing effective adaptive feedback but also triggering proinflammatory pathogenetic effects. In predisposed individuals with neurodegenerative liability, minimal cognitive deficits are already detectable in the early stages of AUD (severe). **Right Panel.** Over time, the pathogenic factors induced by chronic uncontrolled alcohol consumption and the presence of intervening stressors would exacerbate intestinal mucosa damage and gut microbiota dysbiosis, increasing circulating bacterial endotoxins and proinflammatory cytokines, and extending the damage to the liver. The onset of Alcohol-Related Liver Diseases (ARLD) of progressive severity (i.e., steatosis, steatohepatitis, and cirrhosis) would lead to an excess of abnormal metabolites and decreased proteins in the blood, further increasing the overall inflammation and involving other organs, including the brain. In individuals with neurodegenerative liability, cognition progressively worsens over time and imaging will reveal neurodegenerative damage in the brain, leading to the development of more severe alcohol-related neurological disorders, such as *Hepatic Encephalopathy* or *alcoholic dementia*. In individuals with *AUD with Alcohol-Related Clinical Concerns*, the stress response system embedded in limbic-hypothalamic circuits becomes less efficient, with blunted adrenal functions, autonomic overactivation, immune paralysis, and persistent anxiety and depression, contributing to the instability of global homeostatic controls of the Gut Microbiome-Liver-Brain axis functions, ultimately leading to death. The size of the arrows indicates the relevance of pathogenic signals generated and targeted within the Gut Microbiome-Liver-Brain axis, with red indicating high impact. The blue arrows refer to stressors and to stress circuit responses aimed at adaptively reducing the impact of stressors via feedback (i.e., less effective in the right panel).

The majority of data here summarized and discussed are of clinical relevance and in mostly obtained in humans. Our ‘working hypothesis’ is schematically represented in Fig. 1.

## 2 Pathogenic factors engaging the Gut-Liver-Brain axis at different stages of alcohol use disorders

Converging findings indicate that a series of pathogenic processes are already at work in the Gut Microbiome-Brain-Liver axis at the early-stage *AUD*, e.g.*, mild or moderate* (Wang et al., 2010; Day and Kumamoto, 2022), eventually progressing in intensity and duration when the disorder would evolve towards *AUD-severe* and *AUD with Alcohol-Related Clinical Concerns.* Of particular relevance is the condition of *ARLD-cirrhosis with Minimal or Covert Hepatic Encephalopathy,* an evolution due to a mix of pathologic direct effects and adaptive responses occurring at different levels of the axis, well characterised in the literature (Ron and Barak, 2016; Tyler and Leggio, 2024). Several of the pathogenetic mechanisms present in the development from *mild AUD* to *AUD with Alcohol-Related Clinical Concerns* are also triggered by exposure to stressors, as summarized in recent reviews (Foster et al., 2017; Westfall et al., 2021; Mujagic et al., 2022). In the following list, we provide the most agreed pathogenic mechanisms involved in *AUD* that are also produced by exposure to stress, as studied in humans. This list is not exaustive and several reviews are quoted in case of interested for more information on specific topics. The main pathogenic drivers are:

(a) The frequency, amount and duration of exposure to intoxicating levels of alcohol, directly affecting the functioning of neurons, hepatocytes, fibroblasts, gut mucosa epithelial cells, and immune cells (Wood et al., 2013; Arzua et al., 2020; Yan et al., 2023);
(b) Edema, oxidative and mitochondrial damage observed in several brain circuits and produced by the concurrent effects of alcohol, cytokines, hormones, metabolic products, and bacterial endotoxins (Haorah et al., 2008; Song et al., 2014);
(c) Increased inflammation markers in liver and gut tissue (Bishehsari et al., 2017; Osna et al., 2017);
(d) Reduce liver metabolic functioning, leading to blood changes of enzymes, ammonia, bilirubin, albumin, and other biomarkers (Harris et al., 2021; Whitfield et al., 2018);
(e) A genetic risk that contributes to the predisposition for *AUD*, with hundreds of variants that are potentially involved in determining specific sub-phenotypes of *AUD*, and for progressing towards *AUD with Alcohol-Related Clinical Concerns* with neurodegeneration and cognitive impairment (Kranzler et al., 2019; Thomas et al., 2023; Zhou et al., 2023; Zhou and Gelernter, 2024);
(f) The behavioral maladaptive response that engages the individuals with AUD into the “addiction cycle”, in which motivational input (e.g., cue-related alcohol ‘liking’) and negative emotions (e.g., stress, anxiety and depression) are concurring in maintaining the habit of relapsing into excessive alcohol drinking (Sinha, 2022; Tyler and Leggio, 2024);
(g) A series of complex molecular mechanisms that are involved in the neurobiology and plasticity of neural circuits responsible for reward, emotions, stress response, and cognitive control. Detailed knowledge of these mechanisms has accumulated over the last 30 years at an impressive speed (Koob and Bloom, 1988, Gilpin and Koob, 2008, Spanagel et al., 2014, Merlo Pich and Melotto, 2014, Koob, 2021, Heilig, 2023, Volkow et al., 2019). Of particular clinical relevance are: (1) The maintenance of the excitatory-inhibitory balance in these structures, mainly via the glutamate and γ-aminobutyric acid (GABA) neurotransmitter systems; (2) A series of Na+ and Ca2+ channels present on the neuronal membrane controlling neuronal firing; (3) The dopaminergic, noeadrenergic, and serotonergic ascending systems, that are among the critical drivers for reward and stress response; (4) A series of peptidic neurotransmitters, e.g., Corticotropin-Releasing Factor (CRF), Orexin, Enkephalin, Dynorphin, β-Endorphin, Substance P, Neuropeptide Y, Glucagon-Like Peptide (GLP), to name a few, that are pleiotropically involved in arousal, nutrition, emotion, stress, and reward control.
(h) The neuroadaptation occurring in specific human corticolimbic and hypothalamic circuits controlling stress and reward pathways (Koob and Volkow, 2016; Koob, 2021; Fede et al., 2022; Heilig, 2023);
(i) The change of circulating level of stress hormones, i.e., cortisol, adrenaline and noradrenaline (Stephens and Wand, 2012; Roberto et al., 2017; Wentworth and Siragy, 2022);
(j) The increase of gastrointestinal vagal sensitivity and motor reactivity, occurring also in stress (Yu et al., 2020; Leigh et al., 2023);
(k) The increase in permeability of the gut barrier, reported also in stress (Purohit et al., 2008; Leclercq et al., 2012, Leclercq et al., 2014, LaTorre et al., 2023); The changes of the gut microbiome composition, leading to: (1) a predominance of pro-inflammatory strains, defined as dysbiosis, independently observed in alcohol exposures and in stress exposures (DeGruttola et al., 2016; Bajaj, 2019; Westfall et al., 2021, Addolorato et al., 2020); (2) Changes in blood concentrations of metabolic products of gut bacterial origins, normally kept in check by the liver and by the impermeability of the gut mucosa barrier, occurring also in stress (Xu et a. 2022; Ahmed et al., 2022); (3) The increase circulating levels of bacteria endotoxins, such as lipopolysaccharide (LPS) (Purohit et al., 2008; Wang et al., 2010, Leclercq et al., 2012; De Oliveira et al., 2023);
(l) A series of molecular and cellular changes in the immune system produced by chronic exposure to alcohol and/or to stress, affecting the constitutive and adaptive defense systems (Szabo and Saha, 2015; Meredith et al., 2021). In particular we highlight the following features: (1) the increased levels of circulating proinflammatory cytokines (Leclercq et al., 2014b; Marsland et al., 2017; Adams et al., 2020); (2) the enhanced response of immune cells to LPS and other endotoxins via TRL-4 receptors, occurring also in stress (Burnette et al., 2023; Quave et al., 2021); (3) the effects pharmacological blockade of the IL-1b signaling, the NLRP3 inflammasome, and the activity of the Complement system (Erickson et al., 2019); (4) Prolonged stimulation of the immune response system with evolution towards tolerance and immune paralysis observed in *ARDL-steatohepatitis* and *ARDL*-*cirrhosis* (Dhanda and Collins, 2015; Hasa et al., 2022); (5) The abnormal innate immune mechanism signature observed in *AUD with Alcohol-Related Clinical Concerns* that parallels that observed in neurodegenerative disorders with cognitive impairment, i.e., Alzheimer Disease (Ramos et al., 2022).
(m) Alcohol is classified as Group 1 cancerogenic substance and even moderate exposure were found to increase the probability of intestinal organs and breast cancers (Anderson et al., 2023). Exposure to stress has been also considered a possible driver for cancer, possibly via immunological and endocrine mechanisms (Eckerling et al., 2021).

During the early stages of *AUD*, the alcohol-driven triggering of pathogenetic mechanisms has a relatively low impact on the organism, as the stress coping system and cellular defense mechanisms in the Gut Microbiota-Liver-Brain axis remain largely functional. However, over time, prolonged periods of heavy drinking and the establishment of pathological changes in the liver (e.g., steatosis), gut (e.g., increased intestinal permeability), gut microbiota (e.g., dysbiosis) and the brain (neurotransmitter unbalance, neurodegeneration) result in maladaptive responses at behavioral, systemic, and organ/tissue levels. These changes further impair the body's ability to cope with subsequent stressor exposure, that characterise the late stages of the dise.

## 3 Gut microbiota dysbiosis in alcohol use disorder

Recent studies indicate that gut microbiota dysbiosis, a condition associated to (a) overgrowth of pathogens, (b) loss of the overall bacterial diversity, (c) reduction of beneficial bacteria (DeGruttola et al., 2016), occurs in most, if not all, individuals with *AUD*, independently from the presence of an *ARLD* diagnosis. Addolorato et al (2020) showed that decreased alpha diversity, reduction of *Akkermansia* and *Phascolactobacterium* and increase of *Bacteroides* strains were present in the fecal microbiome of individuals with *AUD* with no liver of cognitive impairment, when compared with non-drinker matched healthy subjects. These profiles were sufficient to allow a machine learning-based classification to segregate the individuals into either AUD or healthy groups with an accuracy of 93.4% (Addolorato et al., 2020). This gut microbiome profile was considered of possible pathogenetic relevance, given the high serum levels of LPS and proinflammatory cytokine TNFα, IL-1β, mCP, and IL-6 measured in serum of the same individuals with *AUD* when compared with healthy controls. These observations indicated that gut inflammatory processes and microbiota-originated endotoxemia were already in progress in absence of liver disorders. Another study showed that fecal microbiota changes are already present is individuals with controlled alcohol intake habit, showing a dose-effect relationship in changes of stool consistency and abundance of *Actinobacteria* (Segovia-Rodriguez et al., 2022).

Furthermore, a progressive severity of dysbiosis and associated proinflammatory profile was found in subjects with the worsening of the *AUD with ARLD* conditions*,* from *steatosis into* *steatohepatitis* and *cirrhosis* (Bajaj et al., 2012; Smirnova et al., 2020, Addolorato et al., 2020). A recent study by Llopis et al. (2016) demonstrated that gut microbiota dysbiosis associated to *AUD* contributes to individual susceptibility to ARLD. In their investigation, mice harboring gut microbiota transplanted from individuals with *AUD with ARLD-steatohepatitis* exhibited more severe liver inflammation and necrosis, increased intestinal permeability, and higher bacterial translocation compared to mice with gut microbiota from subjects with *AUD* *without steatohepatitis*. Unfortunately, in this study, no data regarding the brain damage were collected. Accordingly, *AUD* heavy drinkers (<5 units of alcohol daily) *with ARLD-steatohepatitis* displayed a fecal microbiota with changes of abundance of *Ruminococcaceae*, *Veillonellaceae*, *Lachnospiraceae*, *Porphyromonadaceae*, and *Rikenellaceae* strains (Smirnova et al., 2020), while subjects with *AUD with ARLD-cirrhosis* displayed reduced autochthonous taxa, such as *Lachnospiracea, Ruminococcaceae,* and increased *Enterobacteriaceae, Alcaligeneceae*, and *Fusobacteriaceae* (Bajaj et al., 2012). A recent review confirmed the *ARLD-cirrhosis* related reduction of microbial diversity and autochthonous flora (e.g., *Bacteroidetes*, *Ruminococcus*, *Roseburia*, *Veillonellaceae*, and *Lachnospiraceae*) and the overgrowth of potential pathogens (e.g., *Fusobacteria*, *Proteobacteria*, *Enterococcaceae*, and *Streptococcaceae*) (Rocco et al., 2021). Interestingly, in *ARLD-cirrhosis* patients the abundance of *Alcaligeneceae* and *Porphyromonadaceae* was positively correlated with cognitive impairment and associated with endotoxemia and inflammation (i.e., IL-6, TNF-α, IL-2, and IL-13) (Bajaj et al., 2012)*.* In these patients, positive correlations between gut microbiome profiles, blood biomarkers, neuroimaging parameters, and cognitive impairments were further described (Ahluwalia et al., 2016).

The relevance of the gut microbiota profile in determining cognitive impairment was recently assessed using a machine learning algorithm trained on the profile of 20 different fecal microbiome strains: subjects with *ARLD-cirrhosis,* whose cognitive impairment was qualified as *Minimal Hepatic Encephalopathy,* were classified with an 84% specificity vs. subjects *with ARLD-cirrhosis with no cognitive impairment* (Bajaj et al., 2024).

While the relationship between alcohol-related gut dysbiosis and liver-brain damage is well established across the AUD with Alcohol-Related Clinical Concerns specturm, in early stage AUD it is less so. In fact, it is not fully esyablished if dysbiosis is direclty contributing to the maintenance of alcohol addiction, as recently reviewed by Day and Kumamoto (2022), but initial observation are encouraging. Evidence indicates a particular role of gut dysbiosis on alcohol craving and depression. In one study (Leclercq et al., 2014b), individuals with AUD with higher intestinal permeability (leaking gut) and reduced microbiota abundance of *Bifidobacterium* and *Faecalibacterium prausnitzii* showed significantly higher intensity of alcohol withdrawal-associated depression and anxiety than subjects with normal intestinal permeability, also at baseline. In a more recent study, changes in serum kynurenine/tryptophan metabolites of microbiota origin were specifically observed in individuals with AUD vs. control, and they were positively correlated with abundance of the genus *Faecalibacterium* and negatively correlated with depression and alcohol craving scores (Leclercq et al., 2020). Another study in young individuals with AUD, binge drinking was associated with specific microbiome alterations, e.g., reductions of *Alistipes* and increases of *Veillonella*, with craving score correlating with *Bacteroides* spp., *Alistipes* spp.*, Blautia wexlerae*, *Ruminococcus lactaris, Coprococcus euctactus,* among others. Moreover, in the same subjects, greater responsiveness to TLR4 stimulation and increases of IL-6 were also reported, showing a correlation between binge and inflammatory biomarkers (Carbia et al., 2023).

A growing body of data suggest that the gut microbiota and its metabolic products could directly influence the reward system, possibly via interaction with the mesocorticolimbic dopaminergic system (García-Cabrerizo et al., 2021; Dohnalová et al., 2022). Further support comes from studies regarding the gut microbiota composition in mammals, either rodents or humans, with a condition of addiction to opioids or other substances of abuse, showing how gut dysbiosis could affect incentive salience, reward, tolerance, withdrawal, perceived stress, and executive function that characterize these behavioral and clinical conditions (Ren and Lotfipour, 2020, Angoa-Pérez and Kuhn, 2021).

Overall, these data indicate that pathogenic processes in the Gut Microbiome-Liver-Brain axis relevant in *AUD with Alcohol-Related Clinical Concerns*, specifically with *ARLD-cirrhosis* and/or *alcohol-related degeneration of the nervous system*, are already active at the early AUD stages and expressed as mild cognitive impairment and excessive negative emotions & craving, all critical drivers of the Addiction Cycle.

## 4 Brain changes in alcohol use disorder

The earliest manifestation of behavioral and cognitive impairments in individuals with AUD were associated to disruption of brain circuit underlying motivation, working memory, attention, performance monitoring, learning, decision-making and cognitive control (Baler and Volkow, 2006). Several of these deficits of mild-to-moderate intensities were described at the second-third stage of the AUD Addiction Cycle (Kwako et al., 2016; Koob et al., 2020, NIAAA Core Resource on Alcohol, 2024). Binge drinking, defined as a pattern of drinking that brings blood alcohol concentration to 0.08%, in AUD adolescents and young adults, were found to correlate with brain changes consisting of prefrontal cortex thinning and increased neuronal activity in the mesocorticolimbic circuits (Cservenka and Brumback, 2017).

Recent studies exploring the white matter (WM) microstructure using Diffusion Tensor Imaging (DTI) showed a relevant reduction of Fractional Anisotropy (FA) in the telencephalic WM of AUD indivduals with heavy drinking habits (defined as >28 drinks in the past 14 days), particularly within the uncinate fasciculus, a known substrate for executive functions, cognitive control, and memory (McEvoy et al., 2018). Defective DTI subcortical connectivity was also found in individuals with *AUD with ARLD-steatosis* with at least 5 years drinking history. In the same subjects, volumetric MRI showed a reduced gray matter volume in the frontotemporal cortex & subcortical structures, in keeping with results obtained in alcohol-preferring rats (Lanquetin et al., 2021). The functional aspects associated to these structural changes were studied using resting-state fMRI, revealing a defective Default Mode in AUD participants during acute exposure to alcohol, with a marked reduction of the neurocognitive coupling between behavioral performance and fMRI regional signals (Shokri-Kojori et al., 2016). When the AUD condition was associated with liver fibrosis, a significant association with executive function impairments was found, with low thiamine levels and malnutrition being relevant negative modifiers (Ritz et al., 2016).

In case of progression of severe AUD into *AUD with Alcohol-Related Clinical Concerns*, the consistent presence of excessive alcohol drinking, gut microbiome dysbiosis, and leaking guts would affect the functional integrity of the gut, the liver and the brain, significantly contributing to cognitive impairment and compulsive behavior, and eventually neurodegenerative phenomena (Zhar, 2024). Intriguingly, in the general population the occurrence of liver diseases was associated with accelerated age-related decline in total brain and 10.13039/100029390WM volume, further supporting the liver role for brain integrity (Peng et al., 2022). Moreover, epidemiological studies indicate that alcohol misuse and AUD diagnosis were relevant risk factors for all type of dementia (Schwarzinger et al., 2018, Zhar, 2024), while other studies showed a protective effect of occasional or moderate alcohol use (Panza et al., 2012, Mewton et al., 2023).

Overall, the condition of *AUD with Alcohol-Related Clinical Concerns* is strongly associated to the progression into severe neurological and neurodegenerative syndromes, affecting more than 50% of patients with *ARLD-cirrhosis.* The most common syndromes are alcohol-related *Hepatic Encephalopathy*, *Wernicke-Korsakoff syndrome*, and *Alcoholic Dementia* (Zhar, 2024, Zhar, 2024). Briefly, *Minimal (or Covert) Hepatic Encephalopathy* is a condition characterized by mild, but persistent, cognitive impairment affecting executive functions, visuospatial memory and attention. These impairments are assessed using either the Psychometric Hepatic Encephalopathy Score (PHES), the 5-task Repeatable Battery for Assessment of Neuropsychological Status (RBANS), or more recently, the EncephalApp that runs Stroop test on tablet/smartphone (Bajaj et al., 2020). These cognitive deficits were associated to neuroimaging findings, including hyper-densities in the globus pallidus and substantia nigra, reduction of cortical WM volumetry and atrophy in frontotemporal cortex (Zhar, 2024). When *ARLD-cirrhosis* decompensates, episodes of acute *Overt Hepatic Encephalopathy* may occur, with confusion, personality changes, and asterixis, that almost invariably requires hospitalization (Redfield et al., 2024; Gairing et al., 2024). Intriguingly, evolution to dementia was observed with higher frequency in subjects with *ARLD-cirrhosis with Hepatic Encephalopathy* than in subjects with any other kind of cirrhosis (Adejumo et al., 2023). Among other neurodegenerative conditions, *Wernicke Encephalopathy* occurs in 12–18% of subjects whose *AUD with Alcohol-Related Clinical Concerns* is compounded by thiamine deficiency. Untreated, it may progress into *Korsakoff Syndrome*, characterized by marked memory deficits, confabulation, confusion, and erratic behavior (Akhouri et al., 2023). The neuroimaging profile of these disorders consists of a variety of neurodegenerative changes, such as atrophy or hyperintensities affecting mammillary bodies, cortical areas, thalamus, hippocampus, periaqueductal gray matter, dorsal medulla, tectal plates, olivary bodies, and pons (Zhar, 2024; Zhar, 2024). In case of *Alcoholic Dementia,* the main symptom is a frontal cognitive decline, occurring preferentially in socially isolated men under 65 years of age. At variance with other dementia, and in specific of Alzheimer's Disease, *Alcoholic Dementia* is characterized by slow progression and partial reversibility (Zhar, 2024; Zhar, 2024), the main risk factors being thiamine deficiency and iron overload (Listabarth et al., 2020).

However, the damage of brain WM microstructure is not fully reversible. In fact, Fortier et al. (2014) showed that abstinent individuals with AUD with an abuse history of ≥21 drinks per week lasted for 4–5 years and followed by 5 years of sobriety, still displayed widespread bilateral reductions in DTI FA frontal, temporal, parietal, and cerebellar WM tracts, the left inferior frontal gyrus being associated with the drinking severity scores. However, no information was provided regarding the presence of any systemic *Alcohol-Related Clinical Concerns* regarding the Gut Microbioma-Liver axis (Fortier et al., 2014).

Epidemiological data indicates that only a subgroup of individuals with AUD, whose diagnosis was made at younger age, would develop significant neurodegeneratiion later in life. Lifetime diagnosis of AUD ranges between 0.7% and 22.7 % of the general populations. About 90% of these individuals with AUD develop *ARLD-steatosis*, while 10–35% will progress into *ARLD-steatohepatitis,* a serious liver inflammatory condition, and 8–20% will develop *AUD with ARLD-cirrhosis,* generally over several years of alcohol misuse (Ramkissoon and Shah, 2022, Aslam and Kwo, 2023). Regarding the alcohol-related neurological complications, 29–42% of subjects of *AUD with ARLD-cirrhosis* will become affected by *Hepatic Encephalopathy* and 12–24% by *Alcoholic Dementia* (Gairing et al., 2024). Therefore, approximately 5–10% of younger Individuals with AUD are at risk of developing severe alcohol-related neurodegenerative disorders later in life, which is a significant number considering the recent estimate of AUD diagnosis in USA being of 28.9 million (NIAAA, 2024).

The early identification of individuals with AUD who are at risk of developing serious alcohol-related neurodegenerative disorders later in life is of significant interest. Early risk assessment could help to prioritize therapeutic interventions for individuals with ‘prodromal’ or early stage AUD, with the goal of slowing the disorder's progression, improving quality of life, and reducing the economic health burden of society. However, the specific predictive factors for identifying the at-risk AUD subgroup remain unclear.

While some studies have addressed the progression from mild-moderate AUD into more severe AUD or into *AUD with Alcohol-Related Clinical Concerns* specifically affected by *ARLD-cirrhosis*, they have not focused on brain damage or on the worsening of cognitive impairment. A recent study performed on large cohorts in USA showed that the majority of subjects with *severe AUD* diagnosis had a history of *mild-moderate AUD* (Miller et al., 2023), the most relevant predictors being alcohol consumption, conduct disorder, and stress (Colin et al., 2023). The amount of alcohol consumption (i.e., heavy drinking defined as 50 g/day or more) was found to be predictive for the risk of developing *Alcohol-Related Clinical Concerns*, including *ARLD-cirrhosis* (Rehm et al., 2010; Delacôte et al., 2020). The pattern of progression of ARLD-related to alcohol binge or heavy drinking has been known for decades (Tel et al., 1995). More recently, a significant improvement of the predictive logistic model for *AUD with Alcohol-Related Clinical Concerns with ARLD-cirrhosis,* was obtained by including retrospective data that qualified the previous AUD condition, i.e., alcohol consumption, age of onset, duration of hazardous alcohol use, and the presence of variant alleles in the PNPLA3 (patatin-like phospholipase-3) and in the HSD17B13 (hydroxysteroid 17-beta dehydrogenase 13) genes (Miscitelli et al., 2018). In a prospective study on a large cohort of AUD heavy drinkers (>50g/day) in France, the critical risk factors predicting a transition from AUD into *AUD with ARLD-cirrhosis* were gender, BMI-obesity, age-of-onset, alcohol consumption, duration of heavy drinking, and amount of liver fibrosis (Delacôte et al., 2020). Few studies have been focused on the role of cognitive impairment in early stage individuals with AUD as predictor for the clinical outcome over time. For example, Evren et al. (2012) showed that deficits in cognitive control may predict clinical worsening in individuals with AUD when assessed 12 months later, even before the evolution into *AUD with Alcohol-Related Clinical Concerns*.

In other neurodegenerative disorders, such as Alzheimer's Disease, the predictive relevance of mild cognitive impairment occurring at the early stage of disease progression has been established. The FDA strongly recommends targeting prodromal subjects for Alzheimer's Disease with novel therapeutics (FDA guidance documents, 2024). Interestingly, the co-occurrence of chronic alcohol misuse and exposure to stressors was recognized as a relevant risk factor for the worsening of Alzheimer ‘s Disease progression, suggesting a possible overlap in pathogenetic factors (Heymann et al., 2016; Ramos et al., 2022; Seemiller et al., 2024).

In conclusion, while various predictive factors have been identified for AUD progression and related complications, more research is needed to establish robust predictors for the alcohol-related degenerative outcomes in nervous system leading to severe neurological syndromes. The potential role of measurements of cognitive impairment, neuroimaging features, or peripheral biomarkers for neural damage currently considered as early predictors for Alzheimer's Disease warrants further investigation if applied to individuals with AUD at the early stages, to predict *AUD with Alcohol-Related Clinical Concerns of neurological relevance*, given its significance from the disease-modifying treatment perspective.

Here we proposed a working hypothesis to link various aspects of relevance. We hypothesize that certain subgroups of individuals with AUD possess an inherent susceptibility to alcohol induced neurodegeneration. This vulnerability, in combination with environmental stressors, may accelerate the progression towards specific *Alcohol-Related Clinical Concerns of neurological relevance*, including detectable brain damage and behavioral/cognitive changes. We propose that the maladaptive response to alcohol-related pathogenetic mechanisms and intervening stressors occurring in the Gut Microbiome-Liver-Brain axis of individuals with AUD, would serve as the critical drivers of alcohol-related disorder progression.

## 5 Impact of stress on the Gut-Liver-Brain axis in alcohol use disorders

The increased frequency alcohol binge episodes in AUD have been associated to relevant stressful life experiences (Keyes et al., 2011; McCrady and Flanagan, 2021; Sinha, 2022).

Acute alcohol exposure is known to reduce anxiety and negative emotions due to social stressors (Sripada et al., 2011) or distress generated by alcohol withdrawal thanks to its primary actions on limbic structures involved in controlling stress response and reward (Koob & Ventruscolo 2017; Shina, 2022). However, alcohol intake is also associated to a ‘paradoxical’ triggering of the autonomic nervous system and of the Hypothalamus-Pituitary-Adrenal (HPA) axis, resulting in a ‘stress-like’ increased circulating noradrenaline, ACTH and cortisol (Stephens and Wand, 2012; Hwang et al., 2020, Sinha, 2022). Interestingly, the COMBINE study indicated that the levels of alcohol use in individuals with AUD measured at baseline predicted greater-than-typical stress intensity perceived in subsequent weeks, with a stronger and early effects in women, suggesting a cross-sensitization (Witkiewitz et al., 2023). Recent experimental data in rodents indicate that the exposure to stress, or to transient high circulating glucocorticoids, could change the gut microbiome composition (Shukla et al., 2021). In rodents, exposure to psychological stress was seen to impair the barrier function of gut mucosae, in association with a gut microbiome change in bacteria taxa abundance (Geng et al., 2020) and steato-fibrotic liver damage (Xu et al., 2022). In humans, the effects of stress on gut mucosa permeability are still under debate (LaTorre et al., 2023), while an increased prevalence of steatosis/fatty liver disease diagnosis was associated to perceived chronic stress exposure (Kang et al., 2020). Moreover, alcohol and cortisol were seen to act synergistically on the gut microbiota by elevating the abundance of *Enterobacteriaceae* and *Escherichia coli* and reducing the abundance of *Lactobacillus,* effects partially mediated by glucocorticoid receptors expressed in the gut epithelium (Shukla et al., 2022). Studies in rodents showed that the gut microbiome can affect social behavior through the limbic-hypothalamic circuits that mediate stress responses, specifically involving the CRF-dependent activation of the HPA axis (Wu et al., 2021). Adaptive phenomena to mild and acute alcohol intoxication also include the blunting of the endocrine response to external stressors, suggestive of mechanistic interactions between alcohol and stress on the same cellular pathways (Holdstock et al., 2000).

This blunting or tolerance of the adrenal stress response become constitutive in chronic heavy drinking individuals with *severe AUD* (Wentworth and Siragy, 2022), while a sympathetic dominance is indicated by consistent increases of basal heart rate, blood pressure and arousal (Buijs and Van Eden, 2000). These dysregulations would contribute to a reduced efficacy of the integrated defense system against stressors. Regarding the brain, studies in rodent showed that repeated unpredictable stress induce chronic HPA axis dysfunction and persistent abnormal fear memory, liver injury and fibrosis, that was dependent on increased intestinal permeability, changes in the diversity of gut microbiota, and enhanced LPS-dependent TLR4 activation in the liver (Xu et al., 2022; Algamal et al., 2021). This profile shows relevant overlaps with the effects of chronic alcohol exposure, with common mechanisms of actions, as discussed in Chapter 2 (Shukla et al., 2021). This may have a role in determining brain circuit dysfunctions as shown in mice that, when exposed to both chronic stress and alcohol, displayed progressive cognitive deficits (Rodberg et al., 2017). Chronic stress alone could also worsen cognition in ageing humans already affected by mild cognitive impairment, being a concurrent cortisol insufficiency a relevant augmenting factor (Peavy et al., 2009). In a recent Canadian study performed on 3054 middle-aged/elderly subjects, higher Cumulative Life Stress was associated with significant poorer baseline performance in all cognitive tasks, with a faster decline in global cognition in women over time (D'Amico et al., 2023).

A possible interpretation of these findings postulates that the pathogenetic mechanisms triggered by the concurrent exposure to repeated alcohol intake and stressors of various kind would result in a compounded cellular damage within the Gut Microbiota-Liver-Brain axis, progressively eroding the potential of an appropriate stress coping response, eventually contributing to sustain the addiction cycle and accelerating the evolution of AUD into *AUD with Alcohol-Related Clinical Concerns with neurodegeneration and cognitive impairment* (Tripathi et al., 2018, Zahr and Pfefferbaum, 2017; Horrell et al., 2022).

## 6 Therapeutic approaches

The findings so far discussed indicate a likely interaction between stress and a progressively dysfunctional Gut Microbiome-Liver-Brain axis in individuals with AUD, potentially accelerating the progression of alcohol-related disorders towards end-stage conditions characterized by gut dysbiosis, leaking guts, cirrhosis, neurodegeneration and cognitive impairment. Treatments to each of these conditions are available, and others are in development, specifically targeting various mechanisms involved in the Microbiome Gut-Liver-Brain axis. So far, a proper assessment of their integrated effects on disease progression has not been uniformly studied, possibly requiring a novel personalized, patient-centered approach (Leggio and Lee, 2017). Moreover, only few treatments obtained regulatory agencies support to justify their use in the clinics. Here we proposed a list of the most used therapies, and some experimental treatment based on the targeted organ of the Gut Microbiome-Liver-Brain axis; for more extensive presentations and discussions the reader should consult recent reviews (Leggio and Lee, 2017; Bajaj, 2019; Mason and Heyser, 2021; Heilig et al., 2024). Some space will be dedicated to microbiome-based treatments, given the recent advancement in the understanding of this organ system dynamics, its role already present in the *mild-moderate AUD*, and its sensitivity to stress (Cryan et al., 2019; Ahmed et al., 2022).

### 6.1 Treatments for AUD with Brain as a target

Brain circuits and mechanisms underlying alcohol dependence can be targeted with ‘addiction medical treatment’ such as naltrexone, acamprosate or disulfiram, as well as gabapentin, and topiramate considered as ‘additional evidence-based’ medicaments. Baclofen and nalmefene are currently used in Europe for drinking reduction and verenicline, a smoking cessation drug, was also tested with some success (Leggio and Lee, 2017; Mason and Heyser, 2021; McPheeters et al., 2023). All these drugs help in the establishment of abstinence in AUD and showed significant drinking reduction in non-abstinent individuals with AUD by acting on telencephalic, corticolimbic, mesencephalic and hypotalamic circuits involved in reward and negative emotion regulation, as described in several reviews (Koob and Volkow, 2016; Burnette et al. 2022; Mason and Heyser, 2021; Koob, 2021). Regarding their detailed mechanisms of actions, we refer to the informed reveiws from Mason and Heyser (2021) and McPheeters et al. (2023). Briefly, disulfiram acts by chelating copper and zinc, resulting in the blockade of a series of enzymes; when dose daily at 250–500 mg, disulfiram produces an increase of alcohol-induced acetaldheide whose accumulation triggers adversive reactions of nausea, headache, and flushing associated to alcohol drinking. In parallel, the blockade of dopamine β-hydroxylase in the brain reduces the conversion of dopamine to norepinephrine, resulting in increased level of dopamine, whose basal levels are reduced in substance abuse disorders, including AUD (Lanz et al., 2023). Naltrexone and namelfene block opioid μ receptors, attenuating the rewarding effects of alcohol. Acamprosate modulates N-methyl-d-aspartic acid (NMDA) receptor transmission and have indirect effects on the GABA-A receptor neurotrasmitter systems. Gabapentin binds to voltage-gated Ca^2+^channels enhancing the GABA neurotransmission. Topiramate, originally an antiepileptic drug, blocks Na^+^ and Ca^2+^channels, reduces glutamate-mediated neurotransmission and enhanced GABA-A-mediated neurotransmission. Baclofen is active as agonist on GABA-B receptors, while veranicline is a partial agonist on the α4β2 nicotinic receptor that mediate acetylcholine-mediated neurotransmission on the mesocorticolimbic dopaminergic system involved in reward and stress response (Leggio and Lee, 2017, Burnette et al., 2022; Mason and Heyser, 2021).

These drugs are not indicated for the treatments of alcohol withdrawal and should be initiated only following detoxification. In fact, acute withdrawal involves primarily stress-related symptoms that last up to 5 days. In 85% of the cases no medication is needed, but in 15% of cases benzodiazepines are used to quench the acute alcohol withdrawal stressful symptoms (Mason and Heyser, 2021).

These therapeutic interventions are effective in slowing the progression of AUD into *AUD with Alcohol-Related Clinical Concerns* by inducing abstinence or alcohol drinking reduction. In a recent cohort study in AUD patients, the effects of addiction medicine therapy consisting of chronic dosing of naltrexone, gabapentin, baclofen or topiramate, were investigated on the worsening of *ARLD* severity and compared with placebo. Results showed that individuals with AUD that received addiction medicine therapy had a significantly lower risk of developing *ARLD* or worsening the ARLD conditions at study-start. Specifically, those subjects with *AUD with ARD-cirrhosis* that qualified for receiving addiction medicine therapy had lower incidence of hepatic decompensation and improved survival (Vannier et al., 2022, Rabiee et al., 2023). These results should implicate a lower occurrence of *Hepatic Encephalopathy*-related cognitive impairment, but these data were not collected. In fact, only indirect observations are available regarding the possible long-term improvement of residual cognitive impairments in *individuals with AUD with or without ARLD-cirrhosis* made abstinent under addiction medical treatments (Bernardin et al., 2014, Pujol et al., 2018; Butler and Le Foll, 2019). Signals of improvement of cognition in subjects with *AUD with Alcohol-Related Clinical Concerns* were reported with topiramate (Likhitsathian et al., 2012) and acamprosate (Soyka et al., 1998). Intriguingly, abstinence in individuals with AUD was associated with a partial, but significant, reversal of the alcohol-induced dysbiosis (Ames et al., 2020).

Novel research is required to face the large unmet needs still present in AUD, focusing on novel drugs targeting the molecular make-up of brain circuits controlling reward and stress (Koob and Vendruscolo, 2023). Accordingly, a range of innovative treatments for AUD have been recently proposed (Anderson et al., 2019; Heilig et al., 2024), including the Kappa Opioid Receptors antagonist aticaprant, that showed significant antidepressant effects (Schmidt et al., 2024). Aticaprant is believed to act by targeting the ‘reward deficits’ that characterize stress-related disorders and, possibly, the ‘addiction cycle’. The relevance of abstinence and harm reduction for the overall functionality of the Gut-Liver-Brain axis have been recently highlight for the whole area of Substance Use Disorders (SUD) in a series of articles coordinated by Haque and Mellinger (2024), showing that any kind of substance misuse was related with an higher incidence of liver disorders. Therefore, addiction medicine therapeutics are highly recommended in subjects with diagnosis of *AUD- or SUD-related with Liver Disorders* (Robinson et al., 2024).

Another class of potential treatments for AUD are ‘hallucinogenic’ substances. For example, psylocibin targets the same molecular mechanisms in brain circuits also involved in AUD, with minimal side effects (Meinhardt et al., 2021). In a recent clinical trial, psilocybin (25–40 mg) administered in combination with psychotherapy, produced robust decreases in percent of heavy drinking days in individuals with AUD when compared with the control group in active placebo (diphenhydramine 50–100 mg) over the 32 weeks of monitoring (Bogenschutz et al., 2022). Several other studies with psilocybin or derivatives are in progress (Jensen et al., 2022; Vanderijst et al., 2024). While more data are needed, the noteworthy profile of psychedelic drugs as novel treatment of addiction is gaining international interest (Zafar et al., 2023).

Based on preclinical data and empirical clinical observations, GLP-1receptor agonists have been in evaluation as potential treatment in various SUD, including AUD, yielding so far promising results (Chong et al., 2019; Leggio et al., 2023; Chuong et al., 2023; Martinelli et al., 2024). Several trials in AUD (listed on the ClinicalTrial.gov database) are in progress with the GPL-1 agonist exenatide (NCT03232112) and semaglutide (NCT05892432, NCT06546384, NCT06015893, NCT05895643, NCT05520775).

Finally, other non-pharmacological treatments aimed at the maintenance of abstinence are currently in clinical use, including psychosocial or behavioral therapies, e.g., 12-step facilitation, brief interventions, cognitive behavioral therapy or motivational enhancement therapy, to name some of the most used. These approaches to AUD at the early stage, combined with pharmacological interventions, would impact the development of *Alcohol-Related Clinical Concerns,* increasing the probability to induce abstinence or to reduce excessive drinking, and slow down the development of cognitive impairments and, possibly, neurodegeneration (Leggio and Lee, 2017). Managing stress, anxiety, and depression with pharmacological treatments, e.g., serotonin-reuptake inhibitors, could also attenuate the severity of *AUD with ARLD-steatohepatitis or -cirrhosis* and improve the overall Quality of Life (Miller and Raison, 2016; Shaheen et al., 2023). Moreover, treatments targeting the glucocorticoid receptors showed signs of efficacy in both individuals with AUD and rodent models (Koorneef et al., 2018). In one study, the glucocorticoid antagonist mifepristone (600 mg daily taken orally for 1 week) was tested in 56 individuals with AUD as part of a double-blind clinical naturalistic setting and laboratory-based study. Mifepristone showed a substantial reduction in alcohol-cued craving in the laboratory study and reduced alcohol consumption during the 1-week treatment in naturalistic setting (Vendruscolo et al., 2015). Moreover, non-pharmacological mindfulness sessions were seen to improve both the perceived stress and Quality of Life in patient with AUD/cirrhosis (Bajaj et al., 2017a, Bajaj et al., 2017b), However, this latter treatment did not seem to affect the evolution to *ARLD-cirrhosis* or to reduce mortality (et al., 2023).

### 6.2 Treatments for AUD with gut microbiome as a target

As already mentioned, all Guidelines indicate that the most effective treatment for recurrence of the acute episodes of *Overt Hepatic Encephalopathy* consists of the prebiotic lactulose associated with the non-absorbable antibiotic rifaximin. These drugs are active on the gut microbiota and are preferable to other systemic antibiotics to reduce the induction of bacterial resistance in other body districts (Bass et al. 2010, Bajaj et al., 2011). This treatment is presently considered as the Standard of Care for this indication. Other studies showed efficacy of rifaximin in reducing compensated cirrhosis severity and improving cognitive performance in subjects with *AUD with ARLD-cirrhosis and Covert Hepatic Enteropathy* (Bajaj et al., 2011; Nakai et al., 2022). These effects were mediated by changes in the gut microbiota, bacteria gut mucosa translocation, Small Intestine Bacteria Overgrowth, and reduction of the levels of mucin-degrading species, such as *Streptococcus* spp, *Veillonella atypica* and *parvula*, *Akkermansia* and *Hungatella*. Moreover, rifaximin decreases circulatory pro-inflammatory cytokines and improves gut mucosa barrier function (Patel et al., 2022). More recently, in a randomized placebo-controlled trial in individuals with *AUD with mild ARLD*, rifaximin showed a significant reduction of the progression rate of liver fibrosis and of circulating liver enzymes (Israelsen et al., 2023). Intriguingly, during the first months of treatment, the quantity of daily drinking was increased, while the liver pathology improved. This increase of alcohol drinking was transient, relapsing to lower rate within few months. This dissociation argues for a possible gut microbiome mediation in the effect of rifaximin on the liver and, indirectly, on the brain, the latter recovering to a more efficient functioning most likely due to a reduction of bacterial endotoxin and inflammatory signals. In rodents, the gut microbiome was seen to protect the liver from acute alcohol exposure (Chen et al., 2015). Another antibiotic, neomycin, was considered for *Hepatic Encephalopathy* treatment, with inconsistent results (Mandiga et al., 2023); moreover, neomycin adverse events include damages to hearing and kidney, as well as the potential development of antibiotic resistance. Interestingly, rifaximin did not increase the expression of the Antibiotic Resistant Gene (ARG) expression in the gut microbiome (Shamsaddini et a. 2021). A series of studies aimed to alter the response to a drug of abuse were initially performed with ceftriaxone for its off-target property on the glutamate transporter 1 (GLT-1) gene expression in the brain. to later discover that these effects on animal models could have been related to the well-known depleting properties of *β*-lactam antibiotics on the gut microbiota (Rothstein et al., 2005; Rao et al., 2015). In fact, treatments with several antibiotics, including some that do not increase GLT-1 brain expression, reduce relapse-like drinking and alcohol withdrawal severity in animal models, suggesting a primary role for the gut microbiota (Angoa-Pérez and Kuhn, 2021). Moreover, in the literature we could not find human studies with ceftriaxone at doses active on brain GLT-1, and antibiotics are not part of any update reviews on novel or established therapeutics in *AUD with Alcohol-Related Clinical Concerns* (Lee et al., 2024; Heilig et al., 2024).

Other microbiome-targeted therapies for *ARLD* have been proposed including a *Bifidobacterium* and *Lactobacillus* probiotic treatment (Kirpich et al., 2008) or a bacteriophage treatment with cytolysin-positive *E. faecalis* (Duan et al., 2019). Recently, Fecal Microbiota Transplantation (FMT) has gained attention as a potential treatment for gut microbiome-related disorders, being well-established for recurrent *C. difficile* infection (Ooijevaar et al., 2019). In 2017, two pilot studies explored the implementation of FTM from healthy donors in subjects with severe *ARLD-hepatitis* that were steroid-ineligible (Philips et al., 2017) and in *individuals with AUD with ARLD and Hepatic Encephalopathy* (Bajaj et al., 2017b). The latter study showed improved cognition in the FTM group, and the appearance of beneficial taxa in the gut microbiome, with evidence of *Proteobacteria* expansion. In rodent with subacute hepatic damage induced by carbon tetrachloride (CCl4), FMT from human healthy donors demonstrated protective effects vs. CCl4-induced hepatic necrosis and intestinal mucosal barrier damage, improving rat spontaneous behavior and spatial learning abilities. FTM-dependent reduction of systemic and local gut inflammation was associated with reduced TLR4 expression and increased tight junction protein expression, such as Occludin, the gut mucosa (Wang et al., 2017).

In a recent study, individuals with *AUD with ARLD cirrhosis,* already under treatment with lactulose and rifaximin, were transplanted with fecal microbiome from one healthy donor selected for high abundance *Lachnospiraceae* and *Ruminococcaceae*. Enema FMT produced a significant attenuation of craving in 90% of *ARLD-cirrhosis* subjects with AUD diagnosis vs. 30% when exposed to placebo, measured at day 15 from FMT. These results were associated with improved cognition and Quality of Life and lower serum levels of IL-6 and LPS-binding proteins, while the metabolite butyrate/isobutyrate increased only in the FMT group and not in the placebo group (Bajaj et al., 2021). Another randomized placebo-controlled traial performed in 32 advanced *ARLD-cirrhosis* patients confirmed that FMT was a safe and well-tolerated procedure, with no serious adverse events (Shawcross et al., 2023). Some caution, however, is warranted before considering FMT as a standard therapeutic, since its full impact on cirrhosis has not been characterized yet. In fact, in fragile patients with cirrhosis receiving FMT for *C. difficile* gut infection, the occurrence of acute deep hyperammonemic hepatic encephalopathy was reported (Eriksen et al., 2021).

Regarding probiotics, encouraging results came from a preclinical study in rodents using a new strain of *P. pentosaceus*, whose high dose chronic treatments improved alcohol-induced liver damage, gut microbiota dysbiosis, intestinal barrier function, and reduced circulating levels of endotoxin and proinflammatory cytokines (Jiang et al., 2020). Very recently, VE303, a ‘designer’ bacterial consortium planned on key healthy characteristics of their taxa members and originally developed to be used in *C. difficile* infection (Louie et al., 2023), was tested in a randomized placebo-controlled trial in individuals with *AUD with ARLD and Overt Hepatic Encephalopathy* as add-on treatment to lactulose plus rifaximin. The primary endpoints were safety & tolerability, the efficacy endpoint being the psychometric PHES scores measured at 4-week treatment. The results showed a good safety and tolerability profile, with an encouraging trend for improvement of PHES in VE303 vs. placebo (VE303 Topline, 2023). While these results need to be confirmed in Phase III clinical trials, they suggest that a mixture of probiotics rationally selected to compensate for the deficits observed in alcohol-related dysbiosis could improve the clinical outcome of individuals with *AUD with ARLD and Hepatic Encephalopathy*. If confirmed, this approach will support the rationale to move the production of FMT from poorly standardized human donors to highly standardized, quality-controlled, industrial production of consortia mimicking the key aspects of the human healthy microbiota.

Regarding the stress management, indirect evidence stems from the observation that anxiety, depression and poor sleep are common complaints in individuals with *AUD with ARLD-cirrhosis* and -ARLD-*steatohepatitis*, and often resistant to antidepressants (Barboza et al., 2016; Bajaj et al., 2017a, Bajaj et al., 2017b; Shaheen et al., 2023). Improvements of anxiety and depression were reported with a combination of anti-dysbiosis probiotics, as shown for *Lactobacillus helveticus* R0052 and *Bifidobacterium longum* R0175 (De Oliveira et al., 2023). Treatments with prebiotics containing galactooligosaccharides were shown to attenuate HPA axis and negative emotion activation (Schimdt et al., 2015). Some reviews indicate a possible role for probiotic treatments of stress-related disorders (Ma et al., 2021; El Dib et al., 2021). For example, in a recent study in China, *Lactobacillus plantarum* DR7 or placebo were given to 111 stressed adults with moderate stress defined by the PSS-10 questionnaire, for 12 weeks. Significant reduction in plasma cortisol, proinflammatory cytokines and symptoms of stress and anxiety were reported in *Lactobacillus plantarum* DR7 exposed subjects (Chong et al., 2019).

Overall, more confirmatory studies are needed before endorsing these treatments into Guidelines, as recently recommended by a panel of expert (Ma et al., 2021), especially controlling for the impact of stressors in AUD disease severity and progression.

### 6.3 Treatments for AUD with liver as a target

Despite extensive research on the pathophysiology of both *ARLD-steatosis* and non-alcoholic fatty liver disease, no targeted therapies are available on the market. The treatment for *ARLD-steatosis* remains abstinence, body weight control, nutritional support, and corticosteroids or pentoxifylline. As for a similar mild disorder such as the non-alcoholic fatty liver disease, the treatment modality is mainly directed toward control of weight loss, cholesterol blood levels, hypertension, and glycemia via nutritional protocols and weight loss therapeutics, or reduction of parenchymal steatosis and fibrosis (Singh et al., 2017; Kudaravalli and John, 2023). However, several programs for novel therapeutics are in development (An and Sohn, 2023). The most promising are: GLP-1 agonists (Leggio et al., 2023; Nevola et al., 2023), cyclophilin inhibitor, FGF agonists, FXR and PPAR ligands (Tidwell et al., 2023). Regarding the more advanced condition of AUD with *ARLD-steatohepatitis*, corticosteroids such as prednisolone or methylprednisolone are still the most commonly used therapeutics; pentoxifylline is used as an alternative, if steroids are contraindicated (Shaheen et al., 2023). About 20% of these patients will evolve into *ARLD-cirrhosis,* whose form is targeted with several products aimed to reduce the liver damage-born metabolic consequences, including: L-ornithine, L-aspartate, zinc, branched-chain amino acids, non-ureic nitrogen scavengers, acetyl-L-carnitine, and albumin, the liver transplantation being the ultimate therapeutic option (Bjerring et al., 2018; Mangini and Montagnese, 2021; Mandiga et al., 2023). Some of these treatments were episodically reported to produce cognitive improvements. The rising of hyperammonemia is considered a critical trigger for the acute cognitive deficits associated to *Overt Hepatic Encephalopathy*, overall envisioned as an endogenous stressor for the whole Gut-Liver-Brain axis (Gallego-Durán et al., 2024). Currently, hyperammonemia in *ARLD-cirrhosis* is better controlled by gut microbiota targeted treatments (see chapter 5.2).

### 6.4 Integrated treatments for AUD, including digital health

AUD affects approximately 29 million individuals in the United States, with about 2.4 million suffering from *ARLD with decompensated cirrhosis*. However, only about 2% of these patients receive targeted pharmacotherapy for addiction. A recent systematic review suggested that the 'number-needed-to-treat’ to prevent 1 person with *AUD that* reached abstinence from relapsing to drinking was 11 subjects for acamprosate and 18 subjects for oral naltrexone (50 mg) (McPheeters et al., 2023). These figures indicate a significant, but modest, impact of addiction medicine therapy, highlighting room for improvement. Recent data show that prescribing any addiction medicine therapy to *AUD patients* with *decompensated ARLD-cirrhosis* upon hospital discharge can significantly affect disease progression, reducing hospital readmissions by 51% (Bernstein et al., 2024). In the same patient population, a different study found similar reductions in hospital readmissions with a 6-month oral treatment of lactulose and rifaximin, two drugs targeting the gut microbiota (Sanyal et al., 2024). This treatment also reduced the occurrence of *Overt Hepatic Encephalopathy*. Notably, to our knowledge, no study has assessed the combined effects of these two classes of FDA-approved treatments, in subjects with *mild-moderate AUD with ARLD steatosis*, or *no with no ARLD diagnosis,* two conditions that, according with our review, partially share the same pathogenetic mechanisms present in later disorder stages.

Finally, the experience accumulated in the last 10 years in the field of digital health suggests a potential implementation of these technologies to optimize the integrated therapeutic approach (Wu et al., 2022; Sohi et al., 2023; Gananandan et al., 2025) by providing: (a) seamless measurement of biometrics, appetite, cognition and functional activities of daily living; (b) diary, therapy compliance and first aid/support through interactive digital systems; (c) digital therapy, including symptom interpretation, medical advice or psychological support. To date, the key technologies have been: (1) telemedicine and digitally remote-controlled home devices that allow direct and indirect interaction between doctors/psychologists and patients; (2) smartphones and wearable technologies that collect biometric data and allow possible impromptu emergency interaction. These approaches have been beneficial, with improved therapeutic outcomes for both individuals with AUD (Oldham et al., 2024) and *AUD with alcohol-related clinical concerns ARLD cirrhosis* (Gananandan et al., 2025).

However, the recent introduction of generative artificial intelligence (AI) is changing the scenario also in this field (Žigutytė et al., 2024; Narayanan et al., 2024) and promises a new wave of interesting contributions, as generative AI such as ChapGPT is able to integrate very large amounts of data and allow a direct and simple linguistic interactions that could be tailored for the clinicians and for the patients.

## 7 Conclusion

The present review was an attempt to summarize the key facts supporting a working hypothesis based on the construct of Microbiome Gut-Liver-Brain axis as a mechanistic template for supporting novel therapeutic approaches in AUD, here considered in a perspective of disease progression continuum from *mild AUD* to severe *AUD with ARLD and Hepathic Encephalitis or neurodegeneration,* with a particular focus on cognitive impairments. Three conclusive points stand out:

(a) Alcohol drinking, in individuals with AUD, almost immediately affects the gut microbiome, resulting in a dysbiosis that was associated to the occurrence of gut mucosa damage, addictive behavior, cognitive impairments, and liver steatosis in a significant number of drinkers. This condition would further evolve, progressively engaging the organs of the Gut Microbiome-Liver-Brain axis, leading, in some susceptible AUD individuals, to *ARLD*, severe gut leakage, and relevant brain neurodegeneration. However, rigorous studies to support the prognostic value of a specific profile of early dysbiosis and minor cognitive impairment in the developing *AUD with Alcohol-Related Clinical Concerns* with *severe neurological and neurodegenerative* outcomes, need further validation.
(b) In AUD, disease progression depends, at least in part, on the impact of various stressors that would amplify the shared pathogenetic mechanisms already engaged by alcohol exposures in the Gut Microbiome-Liver-Brain axis. One relevant impact is determined by the progressive weakening the adaptive stress copying system present at the cellular, organic, and systemic levels of the Gut Microbiome-Liver-Brain axis occurring in those individuals with AUD transitioning to *AUD with Alcohol-Related Clinical Concerns.* The amount of stress exposure could be of relevance in determining the maintenance of drinking compulsion and the development of more severe ARLD, leaking guts, and cognitive impairments in those AUD individuals at-risk to progress into more severe conditions.
(c) Since a possible neurodegenerative liability could characterized a subgroup of individuals with *mild-moderate AUD* with early gut dysbiosis and minor cognitive impairments, integrated interventions targeting the various levels of the Gut Microbiome-Liver-Brain axis is here proposed as a testable working hypothesis. Accordingly, drugs already in place for *AUD with Alcohol-Related Clinical Concerns* characterized by *ARLD-cirrhosis and Hepatic Enteropathy* affecting the gut microbiota (e.g., lactulose, rifaximin or FMT) could be added on to effective brain-targeting addiction medicine therapeutics (e.g., naltrexone, acamprosate or disulfiram), together with anti-stress interventions. According to the present working hypothesis, this integrated treatment would be more effective on the subgroup of individuals with AUD at-risk for development of severe brain cognitive dysfunctions and neurodegeneration. While here we summarized a series of converging findings supporting this ‘working hypothesis’, there is still a need of defining optimized therapeutic protocols and obtain supportive confirmatory data from clinical studies.

## CRediT authorship contribution statement

**Emilio Merlo Pich:** Writing – review & editing, Writing – original draft, Visualization, Validation, Supervision, Project administration, Methodology, Formal analysis, Conceptualization. **Ioannis Tarnanas:** Writing – review & editing, Writing – original draft, Visualization, Methodology, Conceptualization. **Patrizia Brigidi:** Writing – review & editing, Visualization, Supervision, Formal analysis. **Ginetta Collo:** Writing – review & editing, Supervision, Conceptualization.

## Declaration of competing interest

The authors declare the following financial interests/personal relationships which may be considered as potential competing interests: Emilio Merlo Pich reports a relationship with Alfasigma SpA that includes employment, that ended in 2022. As Gelf Health CEO, Emilio Merlo Pich has been consulting for the following companies: Centessa (UK), Relmada (USA), Novavido (I) and G-Factor (I). The other authors declare that they have no known competing financial interests or personal relationships that could have appeared to influence the work reported in this paper.

## Data Availability

No data was used for the research described in the article.
